# The Effect of Coaching Types on Moral Disengagement in Taekwondo Athletes: The Mediating Role of Pride

**DOI:** 10.3390/ijerph191912306

**Published:** 2022-09-28

**Authors:** Young-Taek Oh, Jun-Phil Uhm, Hyun-Woo Lee

**Affiliations:** Department of Kinesiology & Sport Management, Texas A&M University, College Station, TX 77843, USA

**Keywords:** autonomy-support coaching, controlled coaching, authentic pride, hubristic pride, moral disengagement

## Abstract

The purpose of this study was to examine how coaching styles affect athletes’ moral disengagement. To achieve our objectives, we examined the relationships among perceived coaching types, pride, and moral disengagement in the context of elite taekwondo athletes (*N* = 322). Direct and indirect effects among coaching types, pride, and moral disengagement were assessed through path analysis. The results indicated that the autonomy-support coaching type reduced moral disengagement by decreasing hubristic pride, while the controlled coaching type increased moral disengagement through hubristic pride. Our study found a chain of effects according to the controlled coaching type perceived by taekwondo athletes, hubristic pride, and moral disengagement; therefore, the controlled coaching type and hubristic pride should be closely managed in sport society, as they elicit greater moral disengagement. Managerial strategies to diminish hubristic pride through the autonomy-support coaching type are recommended.

## 1. Introduction

Athletes exert immense effort over substantial portions of their lifetimes to win. In this process, some athletes engage in antisocial behaviors, such as ridiculing opponents, intentionally violating competition rules [1], ridiculing teammates whose behavioral skills are inadequate [2], and deceiving referees [3]. According to studies by Chan et al. [4] and Matosic et al. [5], the coaching style may cause moral disengagement and antisocial behavior in professional athletes. These behaviors are characterized by their negative impact on others. The lingering question is, why does such immoral behavior occur? In order to answer this question, we aim to investigate the immoral behaviors by attributing them to various individual–social variables. Two types of coaching (i.e., autonomy-support coaching and controlled coaching) and two types of pride (i.e., authentic pride and hubristic pride) were examined. Specifically, we examined the relationship of moral disengagement by postulating coaching type and athletes’ pride as antecedent variables.

There is a close relationship between coaches and athletes in sports. Most studies on athletic performance that are currently underway seek links between coaching type, athletes’ social behavior [6], exercise dependence [7], stress [8], and burnout [9]. Pride can be described in two ways: its causes and consequences regarding cognitive, emotional, and behavioral aspects [10]. Authentic pride is founded on self-achievement and represents the pride of self-worth. Hubristic pride, though, is founded on skewed and self-aggrandized self-views rather than self-accomplishments [11]. These two-dimensional concepts of pride have recently been studied in terms of the relationship between pride and moral disengagement [12] and social behavior [13]. However, only these two studies have been conducted thus far [12,13]. Despite evidence pointing to the fact that pride potentially has a strong association with moral behavior toward team members and opponents, this was not pursued further. Researchers have shown a lack of interest in understanding this phenomenon in greater depth [13].

Existing studies have reported that athletes who engage in physical contact, such as soccer players, judo athletes, and wrestlers, exhibit more aggressive behavior in both sports and daily life than those who do not [14,15]. Based on Bredemeier et al. [14,15], we aim to investigate the moral behavior of taekwondo practitioners, a martial art in which physical contact occurs. This is a theoretical way to understand human behavior among martial arts that value politeness as well as to assess whether moral disengagement is derived from an athlete’s individual temperament or whether it can increase or decrease depending on the type of coaching.

Several studies have been conducted on taekwondo athletes, and it has been reported that coaching behavior and social behavior [16], deliberate practice [17], emotional intelligence [18], and sport confidence [19] are closely related. The influence of pride on moral disengagement has also been investigated [13], and it has been suggested that it is imperative to seek an antecedent variable that can control athletes’ pride. Our study tested a broader conceptual model that included coaching styles that could further explain the role of authentic pride in moral behavior, and we concluded that interventions promoting authentic pride and deterring hubristic pride develop greater adaptive morality.

Taekwondo has a cultural characteristic that shows competitive and moral disengagement behavior through its necessity of physical contact between opponents [20]. Examining the effect of coaching types on moral disengagement with two-dimensional pride as a mediator in the context of Korean taekwondo athletes can contribute to academic and practical contexts by expanding self-determination theory and providing coaches with practical implications. While most research has focused on pride among team-sports athletes [12,13], this study focused on taekwondo athletes, which should be a topic of interest and research worldwide for both individual and martial arts. Hence, the purpose of this study is to examine the mediating effect of pride on the relationship between coaching types as perceived by taekwondo athletes and moral disengagement.

### 1.1. Theoretical Background and Hypothesis Development

#### 1.1.1. The Relationship between Coaching Style and Moral Disengagement

Moral disengagement is a manner of social conduct that can predict antisocial behavior [21]. It represents eight psychosocial mechanisms—euphemistic labeling, moral justification, advantageous comparison, diffusion of responsibility, displacement of responsibility, distortion of consequences, dehumanization, and attribution of blame—which are used to minimize negative emotional reactions (e.g., sense of guilt, shame) when engaging in illegal activities. These mechanisms signify a cognitive restructuring of harmful behavior, a minimization of an individual’s responsibility for illegal activities, a distortion of the damage caused by harmful behavior, or a criticism of the victim’s behavior [22]. The initial key point of this research is to identify the antecedents that can reduce or reinforce these mechanisms.

The current study seeks to examine their relevance by establishing the coaching type as an independent variable that can directly affect athletes’ ethical behavior related to sports. According to self-determination theory, a coach’s behavior can be examined through the lens of autonomy-support coaching and controlled coaching [23,24]. The autonomy-support coaching type refers to the coach encouraging athletes to feel comfortable and free to participate in training [25] through an understanding of the strategy or tactic being considered [26]. The controlled coaching type refers to a behavior that values a coach’s opinion more than it values the athletes’ opinions, as characterized by strong pressure and authoritative behaviors toward athletes [27,28]. This has been linked to increasing athletes’ antisocial behavior [16] and psychological frustration [29]. Previous studies on taekwondo [4] noted that athletes exhibited a strong association between moral disengagement and controlled coaching. On this basis, we hypothesized that autonomy-support coaching and controlled coaching have differing effects on moral disengagement.

**Hypothesis** **1** **(H1)** : *A coaching style will affect moral disengagement.*

#### 1.1.2. The Relationship between Coaching Style, Pride, and Moral Disengagement

The second key point of this research is to understand the relevance of moral disengagement and broaden its theoretical extension by postulating pride as a mediator variable. As the association between pride and moral behavior has been gaining attention [13], significant efforts have been made to understand the relevance of moral disengagement in sports among college team sports athletes [30,31]. An arbitrary feeling, such as a sense of guilt, shame, or self-esteem, has recently been used as a variable for ethical behavior regulation.

There are two types of pride: authentic and hubristic [10]. Authentic pride is relational (i.e., it involves engagement with others), indicates a preference for success through assisting or supporting others [10], and has been reported to play a positive role in reducing antisocial behavior [32] and hostility [33]. In contrast, hubristic pride is rooted in conceit, a selfish emotion which reflects arrogance [10] and has been linked to antisocial behavior in everyday life [32] as well as crime and unethical behavior in sports [12].

Our study examined the direct effects of autonomy-support coaching and controlled coaching as perceived by athletes regarding pride and moral disengagement, based on research by Kim et al. [16] and Stanger et al. [13]. We posit that a two-dimensional coaching style and pride will have different effects on moral disengagement. Thus, the following hypotheses were established:

**Hypothesis** **2** **(H2):** *A coaching style will affect pride.*

**Hypothesis** **3** **(H3):** *Pride will affect moral disengagement.*

#### 1.1.3. The Mediating Effect of Pride

There are two aspects to pride, authentic pride, and hubristic pride. Authentic pride is founded on self-achievements and represents the pride of self-worth. This type of pride has been associated with goal regulation, affect, self-control [11], and team identification [33]. Hubristic pride, though, is founded on skewed and self-aggrandized self-views rather than self-accomplishments [12]. This type of pride has been associated with narcissistic self-aggrandizement as well as to aggressive and antisocial behavior [10,11] and moral disengagement [13].

Recent research has shown that pride is related to the dualistic model of passion and moral behavior [12], and the relationship between team identification and supporters’ post-game identity management strategies uses pride as a mediating variable [34]. Likewise, pride has a significant mediating effect between condition and perseverance [35]. There is still scarce research regarding the role of pride between coaching types and moral disengagement in individual sports, particularly in martial arts such as taekwondo. Martial-arts athletes can be evaluated according to personality and attitude in terms of their pride. It is, in other words, as important as their performance. Accordingly, a two-dimensional coaching types for Korean taekwondo athletes mediate pride and has a meaningful influence on moral disengagement, which contributes to the development of sport sociopsychology. Based on previous studies, the following is the research hypothesis:

**Hypothesis** **4** **(H4):** *The relationship between coaching style and moral disengagement will be mediated by pride.*

## 2. Materials and Methods

### 2.1. Participants

The participants in the study included taekwondo athletes from high school, university, and professional teams who were also registered members of the Korea Taekwondo Association in 2022. Participants were recruited using a non-probability purposive sampling method. A total of 322 people were used for the data analysis, with 269 males (83.5%) and 53 females (16.5%). Among them were 72 high school (22.4%), 224 university (69.6%), and 26 professional (8.1%) athletes.

### 2.2. Procedure

#### 2.2.1. Autonomy-Support Coaching

We employed the scale developed by Williams and Deci [35], which consists of nine items and uses a 5-point Likert-type scale. The suitability index score for the confirmatory factor analysis was adequate (χ^2^ = 39.668, d*f* = 19, *p* < *0*.001, *Q* = 2.088, IFI = 0.993, TLI = 0.989, CFI = 0.993, RMSEA = 0.058). The Cronbach’s Alpha value was 0.96.

#### 2.2.2. Controlled Coaching

We used the scale developed by Bartholomew et al. [27], which consists of seven items and uses a 5-point Likert-type scale. The Suitability Index Score for the confirmatory factor analysis was adequate (χ^2^ = 24.052, d*f* = 8, *p* < *0*.001, *Q* = 3.006, IFI = 0.988, TLI = 0.978, CFI = 0.988, RMSEA = 0.079). The Cronbach’s Alpha value was 0.92.

#### 2.2.3. Pride

We used the scale developed by Tracy and Robins [10], which consists of 14 items and uses a 5-point Likert-type scale. The Suitability Index Score for the confirmatory factor analysis was adequate (χ^2^ = 226.552, d*f* = 64, *p* < *0*.001, *Q* = 3.540, IFI = 0.943, TLI = 0.930, CFI = 0.942, RMSEA = 0.089). The Cronbach’s Alpha value was authentic pride 0.90, hubristic pride 0.93.

#### 2.2.4. Moral Disengagement

The study utilized the scale developed by Boardley and Kavussanu [36] was used, which composed of 8 items and uses a 7-point Likert-type scale. The Suitability Index Score for the confirmatory factor analysis was adequate (χ^2^ = 17.208, d*f* = 5, *p* < *0*.001, *Q* = 3.442, IFI = 0.981, TLI = 0.962, CFI = 0.981, RMSEA = 0.087). The Cronbach’s Alpha value was 0.84.

### 2.3. Method of Analysis

The collected data were analyzed using SPSS 24.0 (SPSS Inc., Chicago, IL, USA), SPSS PROCESS Macro 2.13, and Amos 24.0 (IBM, New York, NY, USA) statistical programs with an alpha level of 0.05. First, a frequency analysis was conducted. Second, Cronbach’s alpha values were calculated to check the reliability of each measurement tool, and exploratory factor analysis and confirmatory factor analysis were performed to ascertain the validity of the constructs. Third, Pearson’s product-moment correlation was calculated on major variables. Fourth, SPSS PROCESS Macro [37] was used to explore the mediating effect of pride in the relationship between the coaching type and moral disengagement (Model no. 4). We performed 5000 iterations of bootstrapping to test coefficient estimates including mediation effects and computed bias-corrected 95% percentile intervals.

## 3. Results

### 3.1. Result of Statistical and Correlation Analyses

Descriptive statistics of study variables including mean, standard deviation, skewness, and kurtosis are listed in Table 1. The skewness and kurtosis ranged from −2 to +2 and from −7 to +7 [38], confirming normal distribution (see Table 1). Correlations were performed to examine overall relationships between variables, and all variables were found to be correlated below 0.62 (see Table 1), confirming an absence of multicollinearity [38]. Additionally, composite reliability (CR) and average variance extracted (AVE) were adequate for all measures as reported in Table 1.

### 3.2. Path Analysis of Dimensions of Coaching Type, Pride, and Moral Disengagement

**Figure 1:**
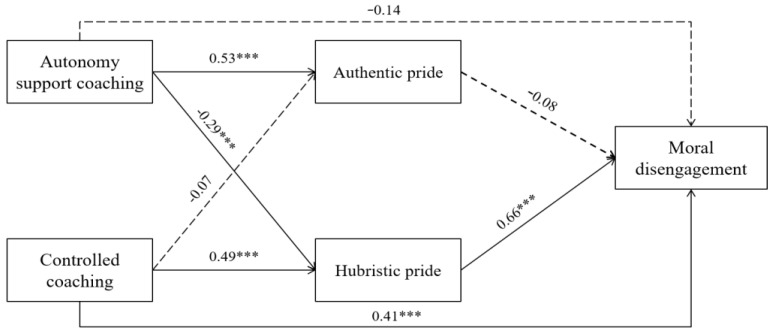
Mediating effect model of pride in the relationship between coaching type and moral disengagement. Note. The values presented in this figure are unstandardized coefficients; The solid line represents significant unstandardized coefficients; dotted line represents non-significant unstandardized coefficients; *** *p* < 0.001.

Figure 1 presents the relationship between coaching type, pride, and moral disengagement (Figure 1). The autonomy-support coaching style had a significant positive effect on authentic pride and a significant negative effect on hubristic pride. A controlled coaching style had a significant positive effect on hubristic pride and a significant positive effect on moral disengagement. As a result of confirming the influence of the mediating variable on the dependent variable, hubristic pride had a significant positive effect on moral disengagement.

### 3.3. Testing Mediating Effects of Pride in the Relationship between Coaching Type and Moral Disengagement

Table 2 shows the results of an examination of the mediating effect of the pride in the relationship between the coaching type and moral disengagement. First, the autonomy-support coaching type has a significant mediating effect on moral disengagement via hubristic pride (95% *CI* = −0.38 to −0.13). On the other hand, there is no mediating effect on moral disengagement via authentic pride (95% *CI* = −0.06 to 0.15). Second, the controlled coaching style has a significant mediating effect on moral disengagement via hubristic pride (95% *CI* = 0.22 to 0.44). On the other hand, there is no mediating effect on moral disengagement via authentic pride (95% *CI* = −0.02 to 0.02).

As for the total indirect effect of coaching style on the dependent variable via the mediating variable, the mediating effects of the autonomy-support coaching style (95% *CI* = −0.37 to −0.05) and the controlled coaching style (95% *CI* = 0.22 to 0.44) were significant.

## 4. Discussion

To explain moral disengagement in the context of sport, we focus on two sporting mechanisms. Ethical justification occurs when harmful behavior is accepted individually or by society as a whole when it is attributed to serving a higher valuable social or ethical purpose [22]. For example, it could refer to an athlete who intentionally injures the opposing athletes to protect their own teammate. On the contrary, shifting responsibility occurs when one’s behavior is perceived to be the result of social pressure or at the behest of another person’s instruction [22]. For instance, in sports scenarios, it refers to an athlete who believes that they are not responsible for injuring the opposing athlete as this action was conducted in association with the coach’s instruction, meaning that the athlete absolves themselves of any responsibility. Research on moral disengagement is being conducted in a variety of contexts.

Examination of the empirical model revealed partial support for all hypotheses. Autonomy-support coaching did not have a significant direct effect on moral disengagement but was found to reduce moral disengagement through the mediation effect by hubristic pride. Controlled coaching was shown to increase moral disengagement with both a direct effect and an indirect effect by hubristic pride. The current research adds an interpersonal aspect to the previously reported relationship between pride and moral disengagement by considering the role of coaching. A framework including the effects of coaching types provides a better understanding of the cognitive process leading to moral disengagement. In addition, previous studies have been conducted primarily on team sport players, but this study expands the boundary condition of self-determination theory by examining the relationship between variables within taekwondo, which is an individual sport.

In an effort to establish a moral framework regarding the dialogue about longevity and sportsmanship, the current investigation into the nature of the precursors affecting moral disengagement can be regarded as a useful research model for academic development. This is in relation to the fields of sport psychology and sociology, as well as for young athletes embarking on their training. Moreover, it is expected that this will be key in terms of informing subsequent studies which are published in the near future. Comparing and contrasting the differences with the existing literature is a useful resource for providing insights for coaching and moral behavior research. The results of this study suggest the promotion of autonomy-support coaching and the avoidance of controlled coaching.

First, meaningful results were yielded after analyzing the direct effect caused by the independent variable. These results were also supported by the following research findings. Transformational leadership that guarantees autonomy and various benefits had a significant effect on pride in a study examining 145 MBA students [4]. A study of 292 athletes found that controlled coaching caused moral disengagement and antisocial behavior through controlled motives [39]. A study found that adolescents who participate in physical activities are at risk of exhibiting moral disengagement behaviors, such as illegal doping and drug consumption [40]. Previous studies have investigated the relationship between pride and moral disengagement. Adding coaching types as antecedent variables indicated significant direct and indirect effects on pride and moral disengagement. The statistically significant findings contribute to a better understanding of athletes’ behavior in sport. It is therefore recommended from a practical standpoint that a more autonomy-supportive style of coaching be employed to reduce athletes’ moral disengagement.

The effect of hubristic pride on moral disengagement was observed as being significantly positive. This is in line with the existing literature. A study of 319 college team athletes revealed a positive effect of hubristic pride on moral disengagement and antisocial behavior [13]. Another study, in this case of paintball players and athletes, found a significantly positive effect of hubristic pride on extreme cheating and immoral behavior [12]. Moreover, the positive effect sizes of moral disengagement on antisocial behavior in sports were moderate to strong [41,42,43,44]. Similarly, it has been proposed that moral disengagement might also be linked to reduced prosocial behavior [21]. Athletes tend to exhibit hubristic attitudes when they view themselves as superior [13], so in this regard, our study highlights the need for coaches to manage the hubristic pride of athletes. Expanding the findings of the current study, we suggest that educational institutions and athletic associations must continuously conduct coaching education by emphasizing the importance of coaching [45].

In other research, an interview with a group of young elite athletes discovered that they occasionally engage in behaviors associated with moral disengagement to justify and minimize individual responsibility for their behaviors as a means of explaining their illegal activities [3]. When athletes undertake moral disengagement behavior, their arrogance causes reputational damage to the wider sport. Previous research findings have suggested that athletes’ arrogance leads them to naturally engage in disrespect or bullying of others while believing that they occupy a special position in society. We argue that such behavior must be addressed by instructors and through coaching. When athletes behave negatively, coaches must evaluate the problem and take proper action [7]. This process will strengthen athletes’ authentic pride and improve the attitude of future athletes.

Having confirmed the mediating effect of pride on the direct effect of coaching type and moral disengagement, it has been supported that employing a coaching style of autonomy support is effective at mediating athletes’ hubristic pride and exerts a significant indirect effect on their moral disengagement. In addition, controlled coaching is observed to have a significant indirect effect on moral disengagement by mediating hubristic pride. These outcomes are supported by other comparable studies revealing a significant mediating effect of pride in the relationship between participative leadership and organizational identification [46]. A study of 1000 individuals involved in sports teams uncovered a significant mediating effect of team pride on satisfaction and a sense of organized citizenship [47]. Pride leads to meaningful consequences as a mediator, and it is a proactive and exemplary attitude in shared activities. This strongly suggests that society should value pride and foster an attitude that seeks authentic pride in its athletic teams. This process indicates the importance of coaching styles in the sports environment. In this respect, coaches must develop the ability to coach not only athletic performance but also personal character, emotional processing, and attitude.

In summary, autonomy support and a controlled coaching style exert different effects on moral disengagement. An autonomy-supportive style suppresses athletes’ moral disengagement by reducing their hubristic pride. The results indicate that a controlled style of coaching increases athletes’ hubristic pride, eventually causing greater moral disengagement. Results indicate that a controlled style of coaching increases athletes’ hubristic pride, eventually causing greater moral disengagement. In this regard, as referenced in the introduction, the first and second key points of this research were addressed effectively. This study is expected to contribute to the sport-psychology literature, as it sheds light on how pride can function as an attribute of athletes’ attitude in sport scenarios. Finally, an athlete’s behavior as affected by coaching may, under some circumstances, represent a socially negative issue. According to our findings, it is critical from a sport psychology and sociological perspective that athletes be coached to exhibit positive attitudes, such as courtesy and respect [48], toward both other members of their sports team and those from the opposing team.

## 5. Conclusions

Among the coaching types experienced by Korean taekwondo athletes, the autonomy-support coaching style had the most positive effect on authentic pride and a significant indirect effect on reducing moral disengagement due to its ability to decrease hubristic pride. On the other hand, the more controlled coaching style caused a high rate of moral disengagement through hubristic pride, implying that this coaching style should be avoided in sports situations.

Despite supporting evidence of relations among new variables via path model analysis, this study presents the following limitations. First, as the cohort of research participants was limited to Korean taekwondo athletes, this study cannot be applied generically to other martial arts. We therefore recommend that further analysis of athletes’ pride based on coaching type be conducted among various martial-arts practitioners in other regions. Second, the proportion of male athletes in this study was greater than 80%; a more balanced gender ratio among participants is essential to any follow-up research. These results can be developed into behavioral types known to elicit athletes’ prosocial behavior due to coaching styles, which should substantially broaden the expansion of theoretical-based dialogue regarding different coaching styles. Finally, more recent research has emphasized the importance of a causal relationship between aggression and moral disengagement [49,50]. The causality between coaching styles, moral disengagement, and aggression should be prioritized for investigation in future research. 

## Figures and Tables

**Table 1:** Correlation Coefficient between Measurement Variables.

Variable	1	2	3	4	5
1. Autonomy-support coaching	1.00				
2. Controlled coaching	−0.45 **	1.00			
3. Authentic pride	0.54 **	−0.09	1.00		
4. Hubristic pride	−0.25 **	0.53 **	−0.17 **	1.00	
5. Moral disengagement	−0.22 **	0.55 **	−0.11	0.62 **	1.00
*M* ± *SD*	4.17 ± 0.81	2.10 ± 0.99	3.69 ± 0.79	2.09 ± 0.92	2.83 ± 1.31
Skewness	−0.79	0.93	−0.07	0.75	0.62
Kurtosis	0.06	0.44	−0.49	0.19	0.28
CR	0.97	0.86	0.91	0.92	0.67
AVE	0.81	0.57	0.59	0.65	0.50

Note. M: mean; SD: standard deviation; CR: composite reliability; AVE: average variance extracted; ** *p* < 0.01.

**Table 2:** Direct and Indirect Effects of Coaching Type, Pride, and Moral Disengagement.

Independent Variable	Dependent Variable	Total Effect	Direct Effect	Total Indirect Effect	Indirect Effect	
					Authentic Pride	Hubristic Pride
		Effect (*SE*) (LL, UL)	Effect (*SE*) (LL, UL)	Effect (*SE*) (LL, UL)	Effect (*SE*) (LL, UL)	Effect (*SE*) (LL, UL)
Autonomy-support coaching	Moral disengagement	−0.35 (0.09) ** (−0.53, −0.18)	−0.14 (0.09) (−0.31, 0.03)	−0.21 (0.08) (−0.37, −0.05)	0.04 (0.05) (−0.06, 0.15)	−0.25 (0.06) (−0.38, −0.13)
Controlled coaching		0.73 (0.06) *** (0.61, 0.85)	0.41 (0.06) *** (0.28, 0.54)	0.32 (0.06) (0.22, 0.44)	−0.01 (0.01) (−0.02, 0.02)	0.32 (0.06) (0.22, 0.44)

Note. LL, UL: bias-corrected 95% confidence interval (lower limit, upper limit); *SE*: standard error; ** *p* < 0.01, *** *p* < 0.001.

## Data Availability

The data presented in this study are available on request from the corresponding author. The data are not publicly available due to privacy issues.
